# Using a ‘one strain-many compounds’ approach to screen a collection of diverse fungi from Aotearoa New Zealand for antibacterial activity against Escherichia coli

**DOI:** 10.1099/mic.0.001641

**Published:** 2026-01-12

**Authors:** Shara van de Pas, Melissa M. Cadelis, Alexander B.J. Grey, Jessica M. Flemming, Duckchul Park, Thomas Lumley, Bevan S. Weir, Brent R. Copp, Siouxsie Wiles

**Affiliations:** 1 Bioluminescent Superbugs Lab, Department of Molecular Medicine and Pathology, Waipapa Taumata Rau – University of Auckland, Auckland, New Zealand; 2 School of Chemical Sciences, Waipapa Taumata Rau – University of Auckland, Auckland, New Zealand; 3 Bioeconomy Science Institute, Manaaki Whenua - Landcare Research Group, Auckland, New Zealand; 4 Department of Statistics, Waipapa Taumata Rau – University of Auckland, Auckland, New Zealand; 5 Te Pūnaha Matatini: Centre of Research Excellence for Complex Systems and Networks, Auckland, New Zealand

**Keywords:** antibacterial, antroalbol H, *Escherichia coli*, fungi, merulin A, minimum inhibitory concentration, screening, steperoxide A

## Abstract

There is an urgent need to identify new chemical compounds with novel modes of action to help manage the antimicrobial resistance crisis. Fungi are prolific producers of secondary metabolites, including those with antimicrobial properties, and contain biosynthetic gene clusters that awaken only under certain growth conditions. In recent years, a wealth of novel fungal biosynthetic pathways and compounds have been identified, suggesting fungi remain a viable source for developing new antimicrobials. The International Collection of Microorganisms from Plants (ICMP) contains thousands of fungi and bacteria primarily sourced from Aotearoa New Zealand. Here, we report the results of our efforts to screen 32 fungal ICMP isolates for activity against *Escherichia coli*, a leading cause of deaths attributable to antimicrobial resistance. We used a ‘one strain-many compounds’ approach, growing the ICMP isolates on seven different media with different pH and various carbon and nitrogen sources. We also tested the isolates for activity at various ages. Our results indicate that several of the tested fungi possess anti-*E. coli* activity and are suitable for further study. Our results also provide further strong evidence for the impact of media on both fungal growth and bioactivity.

## Data Availability

The datasets presented in this study can be found in GenBank as detailed in Table 1 and on Figshare (https://auckland.figshare.com/; doi: 10.17608/k6.auckland.28801247 [1] and doi: 10.17608/k6.auckland.28801265 [2]).

**Table 1.** Fungal isolates used in this study

Fungus	ICMP no.	GenBank accession	Description	Ref
*Agaricales sp*.	17554	MT107903	An undescribed crust fungus in the Cyphellaceae, a family in the phylum Basidiomycota closely related to mushroom species but forming crust or simple hood-like fruitbodies. This isolate was found in Kerikeri, New Zealand, in August 2007.	[19]
*Annulohypoxylon sp*.	18216	MW862791	This fungus in the phylum Ascomycota was cultured in 2009 as a companion of a *Tremella* fungus; it induces a switch from a yeast-like to a mushroom-like form in *Tremella*. It is uncertain if this isolate was isolated from New Zealand or imported.	This study
*Cerrena zonata*	16347	MW862786	*Ce. zonata* is a white rot decay fungus of dead wood that belongs to the phylum Basidiomycota. This isolate was cultured in Ngaruawahia, New Zealand, in April 1995.	[19]
*Coccomyces radiatus*	17340	OP955914	This isolate of a fungus in the phylum Ascomycota was found as a decomposer of fallen leaves of silver beech (*Nothofagus menziesii*) on the West Coast of the South Island of New Zealand in April 1983.	[51]
*Conchomyces bursiformis*	16580	OP955915	A mushroom fungus in the phylum Basidiomycota growing on decayed fallen wood in Te Kuiti, New Zealand, in 2006.	This study
*Cunninghamella echinulate*	1083	MZ325951	*Cu. echinulate* is a common soil saprotroph in the phylum Mucoromycota. This isolate was cultured in Auckland, New Zealand, in December 1978.	[19]
*Epicoccum plurivorum*	17650	MW862790	This species is common as a saprophyte or weak wound parasite on exotic plants in New Zealand. It was cultured from the surface of a mushroom in the Mamaku Plateau, New Zealand, in May 1991.	[19]
*Fomitopsis maire*	16416	OP955916	An endemic polypore fungus in the phylum Basidiomycota associated with a brown rot of living taraire trees (*Beilschmiedia tarairi*). This isolate was cultured from Trounson Kauri Park in Northland, New Zealand, in 1988.	This study
*Helicoon pluriseptatum*	16276	MK432690	A freshwater Ascomycota fungus with unusual helical spores. This isolate was cultured from a dead rimu (*Dacrydium cupressinum*) leaf from Mangamuka Gorge in Northland, New Zealand, in 2004.	This study
*Hohenbuehelia nothofaginea*	16703	OP955917	An endemic mushroom in the phylum Basidiomycota. This isolate was growing on a fallen log of a rimu tree (*D. cupressinum*) in Ruatahuna, New Zealand, in 2005.	This study
*Hyaloscypha spinulosa*	16865	MK432695	Hy. spinulosa is an aero-aquatic species in the phylum Ascomycota. This isolate was cultured from a dead rimu twig (*D. cupressinum*) in Pigeon Bay, New Zealand, in September 2006.	[19]
*Hymenotorrendiella brevisetosa*	18823	JN225946	*Hym. brevisetosa* is a cup fungus in the phylum Ascomycota. This isolate (previously identified as *Torrendiella brevisetosa*) was cultured from beech leaves in Matakitaki, New Zealand, in December 2010.	[19]
*Hypholoma australianum*	21474	MZ325972	*Hyp. australianum* is an orange mushroom with a white stem in the phylum Basidiomycota. This isolate was cultured from wood buried in soil in Otago Lakes, New Zealand, in May 2016.	[19]
*Hypoderma cordylines*	16705	MH682238	*Hypo. cordylines* is an endemic fungus in the phylum Ascomycota. This isolate was found growing on the dead leaves of Cordyline trees in Te Waiiti, New Zealand, in 2004.	[52]
*Laetisaria arvalis*	12896	OP955919	*La. arvalis* is a soil-inhabiting fungus in the phylum Basidiomycota with no mushroom form. This isolate was cultured from dead lawn grass in Auckland, New Zealand, in 1996.	[53]
*Lanzia allantospora*	15649	AY755334	*Lan. allantospora* is an endemic cup fungus in the phylum Ascomycota. This isolate was found on kauri (*Agathis australis*) wood in Northland, New Zealand, in April 1992.	[19]
*Lauriomyces bellulus*	15050	EF029218	*Lau. bellulus* is a saprophytic fungus in the phylum Ascomycota. This isolate was cultured from a dead leaf of *Weinmannia racemosa* in Katikati, New Zealand, in May 2003.	[19]
*Lentinellus pulvinulus*	16586	MW862787	*Le. pulvinulus* is a white rot wood decay mushroom. This isolate was cultured from dead wood in Pehitawa Kahikatea Forest Reserve, New Zealand, in May 2006.	[19]
*Linnemannia elongata*	17447	MZ325962	*Li. elongate* is a fungus in the phylum Mucoromycota. This isolate was cultured from a kauri tree (*A. australis*) in Rotorua, New Zealand, in January 2008.	[19]
*Lophiotrema sp*.	20449	MK432752	This *Lophiotrema* species is an endemic saprobic fungus in the phylum Ascomycota. The isolate was cultured from a living *Phormium cookianum* leaf in Mt. Hutt, New Zealand, in February 2014.	[19]
*Lophodermium culmigenum*	18328	MZ325968	*Lopho. culmigenum* is a plant decay fungus in the phylum Ascomycota. This isolate was cultured from Trounson Kauri Park, Chatham Islands, New Zealand, in November 1992.	[19]
*Mortierella sp*.	20597	MZ325970	This isolate is an unknown species of *Mortierella*, a common soil fungus in the phylum Mucoromycota. This isolate was cultured from rotting wood from Farewell Spit, New Zealand, in May 2014.	[19]
*Mucor laxorrhixus*	20877	MZ325971	*Mu. laxorrhizus* is a saprobe in the phylum Mucoromycota. This isolate was cultured from rotten wood from a stream in St Arnaud, New Zealand, in January 2015.	[19]
*Peniophora lycii*	16714	MZ325959	*Pe. lycii* is a crust fungus in the phylum Basidiomycota. This isolate was cultured from decaying wood in Te Waiiti, New Zealand, in May 2001.	[19]
*Pleohelicoon richonis*	16226	MK432689	*Pl. richonis* is an aquatic fungus in the phylum Ascomycota. This isolate was cultured from Horseshoe Lake Reserve, Christchurch, New Zealand, in 2005.	This study
*Rigidoporus concrescens*	18193	OP955918	*R. concrescens* is a polypore fungus in the phylum Basidiomycota, which causes the disease ‘white-pocket heart rot’ on native New Zealand trees. This isolate was cultured from a kauri tree (*A. australis*) in Northland, New Zealand, in 1995.	This study
*Sordariomycetes sp*.	16864	MZ325960	This is an unknown *Sordariomycetes* species with <90 % match to other isolates. It was cultured from a dead rimu twig (*D. cupressinum*) in Pigeon Bay, New Zealand, in September 2006.	[19]
cf. *Spirosphaera sp*.	20907	MK432784	This isolate is an unknown species of *Spirosphaera*, an aquatic hyphomycete genus in the phylum Ascomycota, with spores in loosely interwoven, branched, septate coils. This isolate was cultured from dead wood in a stream in St Arnaud, New Zealand, in 2015.	[54]
*Trametes coccinea*	13182	MW862784	*Tra. coccinea* is a wood decay bracket fungus in the phylum Basidiomycota. This isolate was cultured from a dead radiata pine in Northland, New Zealand, in September 1985.	[19]
*Trechispora stevensonii*	17555	MZ325964	*Tre. stevensonii* is a crust fungus in the phylum Basidiomycota. This isolate was found on the surface of an exotic pine tree in Riverhead Forest, New Zealand, in 2007.	This study
*Umbelopsis ramanniana*	17492	EU770239	*U. ramanniana* is a saprobe in the phylum Mucoromycota. This isolate was cultured from grapevines in Whenuapai, New Zealand, in April 2007.	[19]
*Xylariaceae sp*.	16006	MZ325954	This isolate is an unknown genus and species of the *Xylariaceae* family in the phylum Ascomycota cultured from Ahuriri Reserve, Christchurch, New Zealand, in May 2005.	[19]

## Introduction

It has been 80 years since Sir Alexander Fleming received the Nobel Prize in Physiology or Medicine for discovering the antibiotic penicillin. Penicillin and other antibiotics have become crucial medical tools in the intervening years. As well as being given to treat those with a bacterial infection, antibiotics are also used to prevent infection in vulnerable patients, including people undergoing chemotherapy or surgery. In his Nobel Lecture in December 1945, Fleming warned that it was ‘not difficult to make microbes resistant to penicillin’ and advised against using the drug negligently [3]. Since then, resistance to antibiotics and other antimicrobials has become a major threat to human health around the world [4], exacerbated even further by the coronavirus disease 2019 pandemic [5–7].

In 2019, antibiotic-resistant bacteria were responsible for 1.27 million deaths globally, with *Escherichia coli* identified as the leading causative agent [8]. While the share of deaths caused by *E. coli* varied by region, in the high-income super-region, this bacterium alone was linked to almost one in four deaths attributable to antimicrobial resistance [8]. To help manage this crisis, there is an urgent need to identify new chemical compounds with novel modes of antimicrobial action [9, 10]. Most antibiotics in the clinic today derive from compounds identified from soil microbes, beginning with the discovery of penicillin from the fungus *Penicillium* [11]. In recent years, a wealth of novel fungal biosynthetic pathways and compounds have been identified [12–17], suggesting fungi remain a viable source for developing new antibiotics.

Manaaki Whenua, a Crown Research Institute in Aotearoa New Zealand, is the custodian of the International Collection of Microorganisms from Plants (ICMP), which contains thousands of fungi and bacteria primarily sourced from Aotearoa and the South Pacific [18]. While the collection includes some fungal genera traditionally used for antibiotic production, it has not been rigorously tested for antimicrobial activity. We have previously reported the results of our efforts to screen a small portion of the collection for anti-mycobacterial activity [19]. Of relevance, we identified several ICMP fungal isolates with activity against *Mycobacterium abscessus* and *Mycobacterium marinum*, including an unknown species of *Boeremia* and an isolate of a novel genus and species in the family Phanerochaetaceae [19].

Here, we use a ‘one strain-many compounds’ (OSMAC) approach [20, 21] – growing the fungi on seven different media – to screen 32 ICMP isolates for activity against *E. coli*. Our results indicate that several of the tested fungi possess anti-*E. coli* activity and are suitable for further study.

## Methods

### Bacterial strains and growth conditions

In this study, we used a bioluminescent derivative of the antibiotic-testing strain *E. coli* ATCC 25922, designated 25922 lux [22], expressing the bacterial luciferase (*lux*) operon from the integrating plasmid p16Slux [23]. We grew *E. coli* 25922 lux cultures in Mueller Hinton media (Fort Richard, New Zealand) at 37 °C, shaking at 200 r.p.m. as necessary.

### Fungal material and growth conditions

Fungal isolates (Table 1) were provided by Manaaki Whenua – Landcare Research Group, the New Zealand research organisation responsible for curating the ICMP. As previously described, we stored fungal isolates individually in cryotubes at −80 °C [19]. Briefly, we made freezer stocks by growing each fungus on 1.5% potato dextrose agar (PDA) at room temperature (~20 °C) and excising small cubes of agar (5–6 mm in length) from the fungus’ growing edge. We placed these cubes within a cryovial containing 1 ml of 10% glycerol and rested them for 1 h, after which we removed the remaining liquid glycerol and stored the tubes at −80 °C.

### Fungal DNA extraction and ITS sequencing

As previously described [19], we used a small portion of mycelium from growing fungi and extracted DNA using the REDExtract-N-Amp^™^ Plant PCR ReadyMix (Sigma-Aldrich, New Zealand) according to the manufacturer’s protocol. Briefly, we diluted DNA samples fivefold and amplified them using the ITS1F (5′ CTTGGTCATTTAGAGGAAGTAA 3′) and ITS4 (5′ TCCTCCGCTTATTGATATGC 3′) primer set in a 10 µl reaction. We used the following PCR conditions: initial denaturation at 94 °C for 3 min, followed by 40 cycles of denaturation at 94 °C for 30 s, annealing at 52 °C for 30 s and extending at 72 °C for 30 s. The final extension was performed at 72 °C for 7 min. We checked the amplified DNA by gel electrophoresis before sequencing using an Applied Biosystems^™^ 3500xL Genetic Analyzer using ITS1F and ITS4 primers. We trimmed and combined the sequence data using Geneious Prime (Biomatters, New Zealand), removed any low-quality reads and used blast to verify fungal identification. Optimized sequence data were aligned using MEGA12 [24]. A phylogenetic tree was built from aligned sequences using the neighbour-joining and Tamura–Nei genetic distance models in Geneious (v. 21.0.7, Biomatter Inc.). The tree was constructed by comparing ITS sequences to those in GenBank, and consensus sequences were assembled *de novo* using the Geneious assembler.

### Primary fungal screening using the zone of inhibition assay

**Fig. 1.**
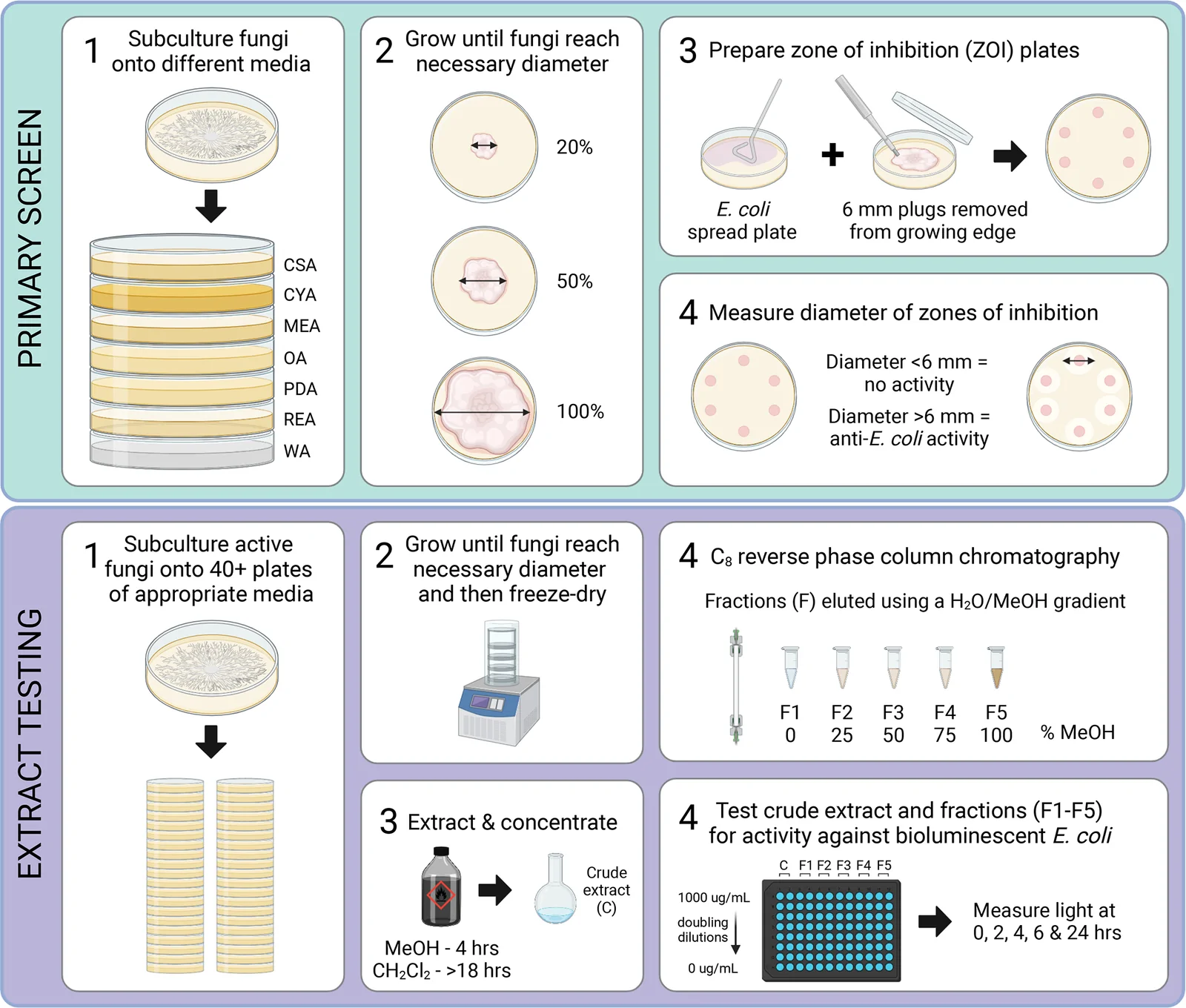
Schematic of experimental workflow. Schematic produced using Biorender.com.

We first grew each fungal isolate on PDA. Once grown, we excised 1 cm cubes from the growing edge using a scalpel and placed single cubes onto the centre of 90 mm Petri dishes containing either PDA or one of six other growth media: Czapek solution agar (CSA), Czapek yeast extract agar (CYA), malt extract agar (MEA), oatmeal agar (OA), rice extract agar (REA) and water agar (WA). We measured the diameter of each fungus at regular intervals over 60 days (Fig. 1). All fungal isolates were incubated at room temperature. Cultures were grown in semi-darkness, as while the laboratory is permanently lit, Petri dishes were stored stacked in plastic boxes under laboratory benches.

Where possible, we tested each fungus for anti-*E. coli* activity once they had grown to cover 20, 50 and 100% of a 90 mm Petri dish. We used a modified version of the zone of inhibition (ZOI) assay, which we have published on the protocol repository website protocols.io [25]. Briefly, overnight cultures of *E. coli* 25922 lux were diluted in Mueller–Hinton broth II (MHB) to an OD at 600 nm (OD_600_) of 0.01 (the equivalent of ~10^6^ bacteria per ml) and used to inoculate CSA, CYA, MEA, OA, PDA, REA and WA plates with a lawn of *E. coli*. Using a 6 mm punch biopsy tool (Paramount, Capes Medical, New Zealand), we removed plugs from the growing edge of each fungus and placed these fungi side down onto the corresponding *E. coli* lawn plates. We incubated these plates overnight at 37 °C, after which we measured the diameter of any ZOI (Fig. 1). We performed these assays using at least three independent biological replicates of each fungus.

### General chemistry conditions

We recorded NMR spectra using a Bruker Avance DRX-400 spectrometer or an Avance III-HD 500 spectrometer operating at 400 or 500 MHz for ^1^H nuclei and 100 or 125 MHz for ^13^C nuclei utilizing standard pulse sequences at 298 K. We recorded high-resolution mass spectra on a Bruker micrOTOF QII (Bruker Daltonics, Bremen, Germany). We carried out analytical TLC on 0.2 mm thick plates of DC-plastikfolien Kieselgel 60 F254 (Merck, New Zealand). We carried out reversed-phase column chromatography on C_8_ support with a pore size of 40–63 µm (Merck, New Zealand). We performed gel filtration chromatography on Sephadex LH-20 (Pharmacia, New Zealand). We carried out flash chromatography on diol-bonded silica with a pore size of 40–63 µm (Merck, New Zealand). We used solvents of analytical grade or better and/or purified according to standard procedures.

### Fungal growth and extraction

We grew fungal cultures on solid media at room temperature and then freeze-dried them (Fig. 1). We extracted the dry cultures with MeOH (Sigma-Aldrich, New Zealand) for 4 h, followed by CH_2_Cl_2_ (Sigma-Aldrich, New Zealand) overnight. We concentrated the combined organic extracts under reduced pressure. We subjected the crude extracts to C_8_ reversed-phase column chromatography eluting with a gradient of H_2_O/MeOH (Sigma-Aldrich, New Zealand) to afford five fractions (F1–F5) (Fig. 1). We have published our protocol on the website protocols.io [26]. Selected fractions underwent further purification. Full details are provided in Table S1, available in the online Supplementary Material.

### MIC testing of fungal extracts

We have published a detailed description of our method on the protocol repository website protocols.io [27]. Briefly, overnight cultures of *E. coli* 25922 lux were adjusted to a final concentration of ~5×10^5^ cells ml^−1^ in MHB (Fort Richard, New Zealand). We dissolved the fungal fractions in DMSO (Sigma-Aldrich, New Zealand). We added these in duplicate to the wells of a black 96-well plate (Nunc, Thermo Scientific) at doubling dilutions with a maximum concentration of 2,000 µg ml^−1^ (Fig. 1). We then added 50 µl of diluted *E. coli* 25922 lux to each well, giving final extract concentrations of 0–1,000 µg ml^−1^ and a cell density of ~10^5^ c.f.u. ml^−1^. We used the antibiotic erythromycin (Sigma-Aldrich, New Zealand) as a positive control at 250 µg ml^−1^. We measured bacterial luminescence at 0, 2, 4, 6 and 24 h using a Victor X-3 luminescence plate reader (PerkinElmer) with an integration time of 1 s. Between measurements, plates were covered, placed in a plastic box lined with damp paper towels and incubated with shaking at 100 r.p.m. at 37 °C. We have defined the MIC as causing a 1 log reduction in light production, as previously described [28, 29].

### Antimicrobial testing of pure compounds

Antimicrobial evaluation of the pure compounds against *Acinetobacter baumannii* ATCC 19606, *Candida albicans* ATCC 90028, *Cryptococcus neoformans* ATCC 208821, *E. coli* ATCC 25922, *Klebsiella pneumoniae* ATCC 700603, *Pseudomonas aeruginosa* ATCC 27853 and methicillin-resistant *Staphylococcus aureus* ATCC 43300 (MRSA) was undertaken at the Community for Open Antimicrobial Drug Discovery (CO-ADD) at The University of Queensland (Queensland, Australia) according to standard protocols [30].

Colistin and vancomycin were used as positive bacterial inhibitor standards for Gram-negative and Gram-positive bacteria, respectively. Fluconazole was used as a positive fungal inhibitor standard for *C. albicans* and *Cr. neoformans*. The quality control of the assays was determined by the antimicrobial controls and the Z’-factor (using positive and negative controls). Each plate was deemed to fulfil the quality criteria if the Z’-factor was above 0.4, and the antimicrobial standards showed a full range of activity, with total growth inhibition at their highest concentration and no growth inhibition at their lowest concentration [30].

### Statistical analysis

After confirming that residuals were approximately normally distributed and checking that a random intercept was sufficient, we fitted mixed ANOVA models for log (size) with a random intercept for biological replicates within organisms. Two observations of zero size were omitted. We tested the main effects and second-order interactions of the variables using the lme4 and car packages in R [31–33].

## Results

### Growth characteristics of a panel of ICMP fungal isolates

**Fig. 2.**
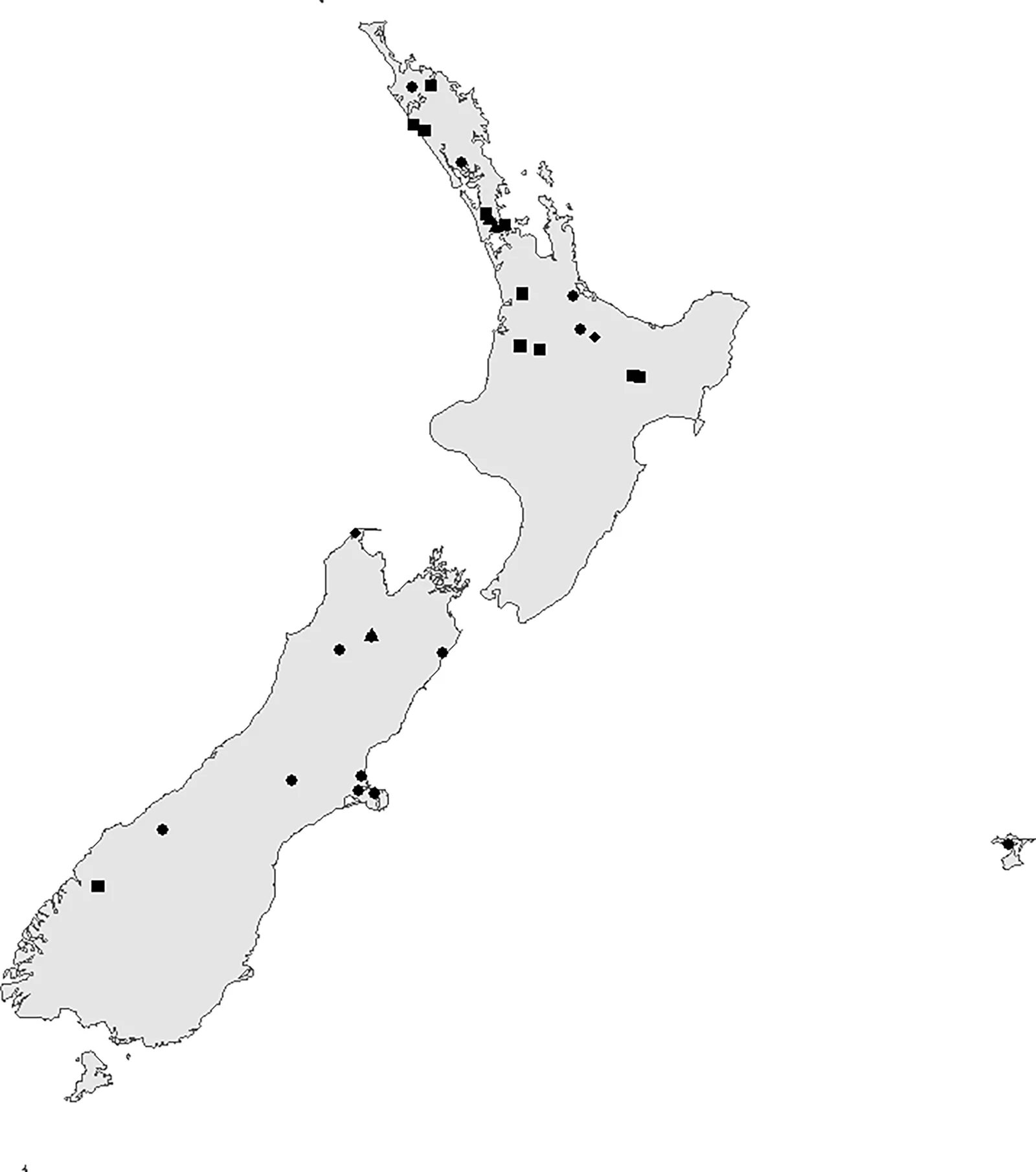
Geographical spread of fungal isolation locations within the archipelago of Aotearoa New Zealand. Key: Ascomycota are shown as filled circles, Basidiomycota are shown as filled squares, Mortierellomycota are shown as filled diamonds and Mucoromycota are shown as filled triangles.

**Fig. 3.**
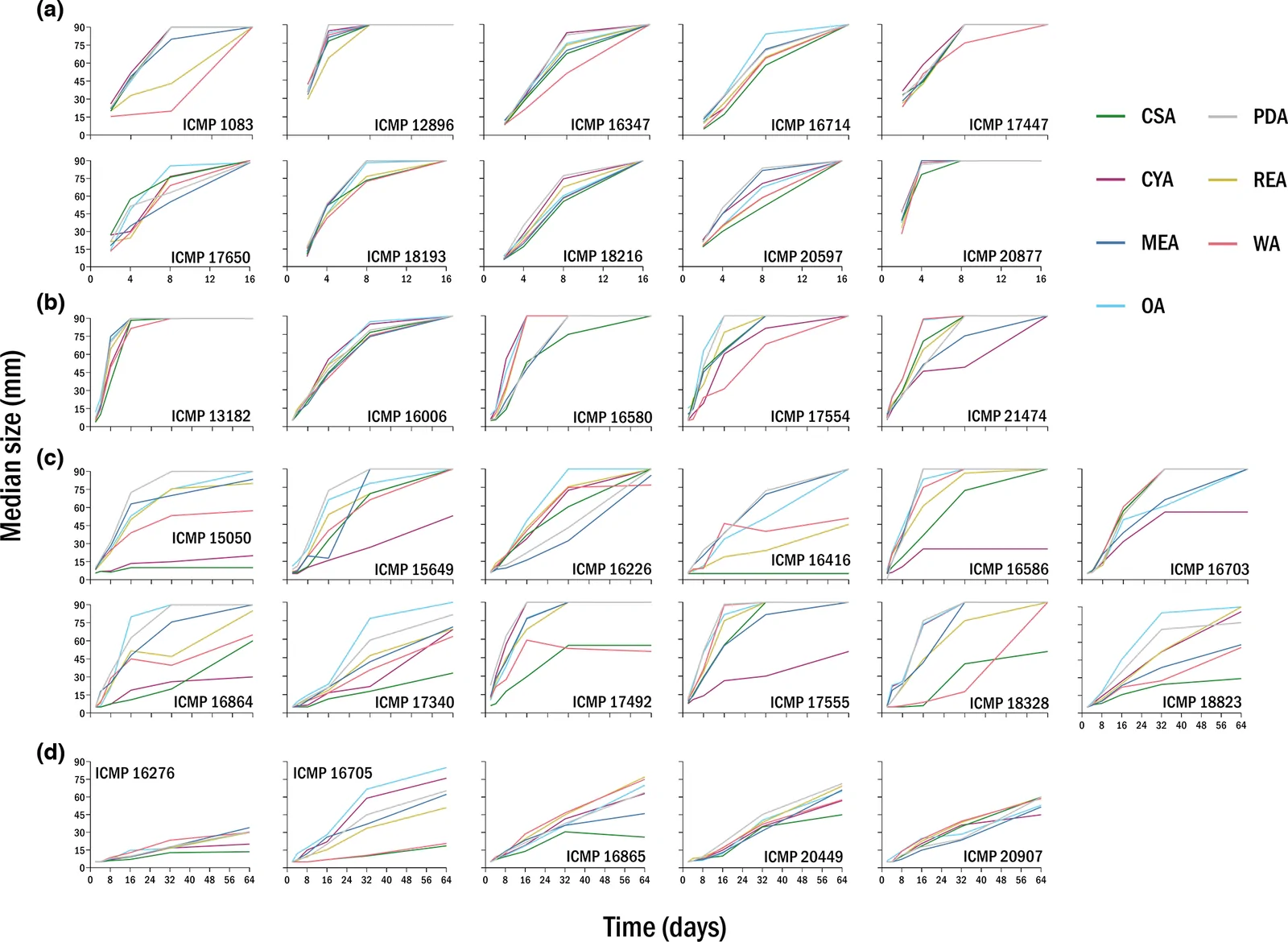
Growth of ICMP isolates on seven different media. Data are presented as median size in mm of at least three biological replicates. ICMP isolates arranged as (**a**) those that covered a 90 mm Petri dish by 16 days regardless of media type, (**b**) those that covered a 90 mm Petri dish by 60 days regardless of media type, (**c**) those that covered a 90 mm Petri dish by 60 days when grown on at least one media and (**d**) those that did not cover a 90 mm Petri dish by 60 days when grown on any of the seven media. Media: Czapek Solution Agar (CSA); Czapek Yeast extract Agar (CYA); Malt Extract Agar (MEA); Oatmeal Agar (OA); Potato Dextrose Agar (PDA); Rice Extract Agar (REA); Water Agar (WA).

We assessed the growth of 32 ICMP isolates, collected between 1978 and 2016 (Table 1) from locations across Aotearoa New Zealand, including the North, South and Chatham Islands (Fig. 2), on seven different media: CSA, CYA, MEA, OA, PDA, REA and WA, measuring the diameter of each fungus at regular intervals for up to 60 days (Fig. 3). The physical characteristics (form, elevation, margin and colour) of each isolate when grown on the different media are provided in Table S2.

We observed that the ICMP isolates could be divided into four categories: (1) those that grew to entirely cover a 90 mm Petri dish by 16 days regardless of the growth medium [10/32 (31.3%)] (Fig. 3a); (2) those that grew to entirely cover a 90 mm Petri dish by 60 days regardless of the growth medium [5/32 (15.6%)] (Fig. 3b); (3) those that grew variably, entirely covering a 90 mm Petri dish by 60 days when grown on at least one medium [12/32 (37.5%)] (Fig. 3c); and (4) those that did not entirely cover a 90 mm Petri dish by 60 days when grown on any of the seven media [5/32 (15.6%)] (Fig. 3d). The Mucoromycota were over-represented in the first category, with 4/5 of the isolates able to grow rapidly on all the media tested, while the Ascomycota were over-represented in the fourth category (5/5), comprising those ICMP isolates that did not grow to cover a 90 mm Petri dish within 60 days on any media.

We calculated the growth rate (as mm day^−1^) of each ICMP isolate when actively growing on each of the seven media (Table 2 and Fig. 3). We observed strong statistical evidence of differences in growth rate between phyla (*P*=0.000125) and between media (*P*≤2.2×10^−16^) and of interactions between phylum and medium (*P*≤2.671×10^−16^). Some isolates grew as slowly as 1 mm day^−1^ on some media (for example, *Fomitopsis maire* ICMP 16416, *Helicoon pluriseptatum* ICMP 16276, *Hypoderma cordylines* ICMP 16705 and *Lophodermium culmigenum* ICMP 18328), while others grew at a rate of more than 20 mm day^−1^ (*Laetisaria arvalis* ICMP 12896 and *Mucor laxorrhixus* ICMP 20877) (Table 2). On average, the Mucoromycota isolates grew faster than the Basidiomycota isolates, which were faster than the Ascomycota. Median growth rates for the Mucoromycota isolates ranged from 8.1 to 12.9 mm day^−1^, depending on the growth medium, while for the Basidiomycota and Ascomycota, they were 3.8 to 5.9 and 1.2 to 1.4 mm day^−1^, respectively (Table 2).

**Table 2.** Fungal growth rates (mm day^−1^) on various solid media

Media*	All ICMP isolates (*n*=32)			Ascomycota (*n*=15)			Basidiomycota (*n*=12)			Mucoromycota (*n*=5)		
	Min.	Max.	Median	Min.	Max.	Median	Min.	Max.	Median	Min.	Max.	Median
CSA	0.7 (ICMP 16705)	19.5 (ICMP 12896)	2.4	0.7 (ICMP 16705)	11.8 (ICMP 17650)	1.2	0.9 (ICMP 16416)	19.5 (ICMP 12896)	3.8	2.2 (ICMP 17492)	19.5 (ICMP 20877)	10.8
CYA	0.8 (ICMP 16276)	23.3 (ICMP 20877)	2.7	0.8 (ICMP 16276)	9.6 (ICMP 17650)	1.2	0.9 (ICMP 16416)	19.4 (ICMP 12896)	3.8	6.9 (ICMP 17492)	23.3 (ICMP 20877)	12.9
MEA	0.8 (ICMP 16276)	19.8 (ICMP 20877)	3.5	0.8 (ICMP 16276)	7.9 (ICMP 17650)	1.3	2.5 (ICMP 16580)	18.2 (ICMP 12896)	4.2	6.0 (ICMP 17492)	19.8 (ICMP 20877)	10.9
OA	0.9 (ICMP 16276)	19.6 (ICMP 12896)	4.7	0.9 (ICMP 16276)	9.2 (ICMP 17650)	1.6	1.6 (ICMP 16416)	19.6 (ICMP 12896)	5.9	5.1 (ICMP 17492)	18.6 (ICMP 20877)	11.2
PDA	0.8 (ICMP 16276)	22.8 (ICMP 20877)	4.0	0.8 (ICMP 16276)	10.7 (ICMP 17650)	1.5	2.4 (ICMP 16580)	19.8 (ICMP 12896)	5.5	9.4 (ICMP 17492)	22.8 (ICMP 20877)	11.6
REA	0.8 (ICMP 16276)	16.4 (ICMP 20877)	3.5	0.8 (ICMP 16276)	9.1 (ICMP 17650)	1.4	1.4 (ICMP 16416)	15.2 (ICMP 12896)	4.8	5.6 (ICMP 17492)	16.4 (ICMP 20877)	8.3
WA	0.7 (ICMP 18328)	20.5 (ICMP 12896)	3.7	0.7 (ICMP 18328)	7.6 (ICMP 17650)	1.2	1.7 (ICMP 16416)	20.5 (ICMP 12896)	4.9	4.7 (ICMP 17492)	13.9 (ICMP 20877)	8.1

Key: *Czapek Solution Agar (CSA); Czapek Yeast Extract Agar (CYA); Malt Extract Agar (MEA); Oatmeal Agar (OA); Potato Dextrose Agar (PDA); Rice Extract Agar (REA); Water Agar (WA).

### Whole-cell screening identified many ICMP fungal isolates as having anti-*E. coli* activity

**Fig. 4.**
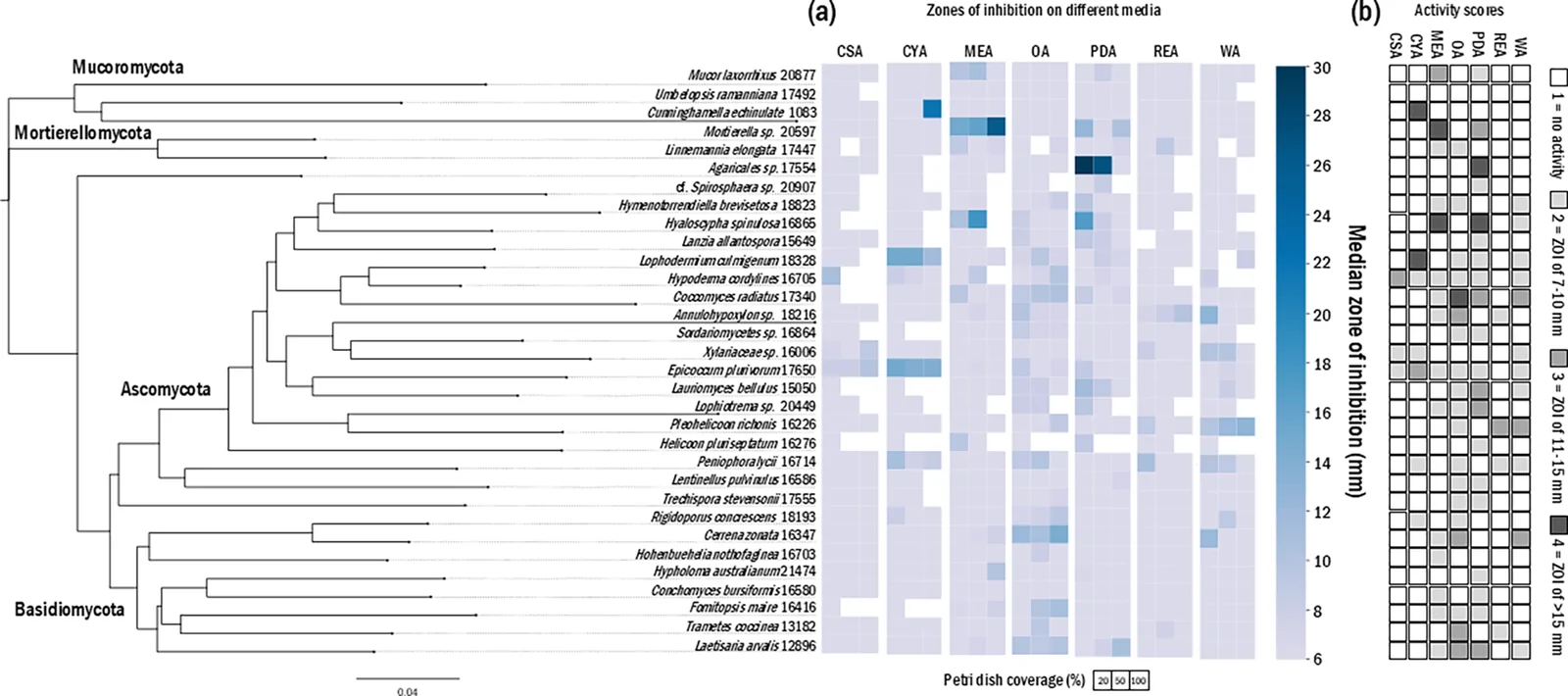
Phylogeny of ICMP isolates and their activity against *E. coli* when grown on different media. Key: Isolates were tested for activity against *E. coli* ATCC 25922 lux in ZOI assays when they had grown to cover 20, 50 and 100% of the area of a Petri dish. Data are presented as (**a**) median ZOI (mm radius) of at least three biological replicates and (**b**) as median ZOI scores. A score of 0 represents no activity, a score of 1 equates to the production of zones with a diameter of 7–10 mm, a score of 2 with zones with diameters of 11–15 mm and a score of 3 to zones with a diameter of >15 mm. Media: Czapek Solution Agar (CSA); Czapek Yeast extract Agar (CYA); Malt Extract Agar (MEA); Oatmeal Agar (OA); Potato Dextrose Agar (PDA); Rice Extract Agar (REA); Water Agar (WA). The phylogenetic tree was constructed in Geneious Prime (Biomatter Inc.) using the neighbour-joining and Tamura–Nei genetic distance models. The tree was constructed by comparing ITS sequences to those in GenBank, and consensus sequences were assembled *de novo* using the Geneious assembler.

We screened 32 ICMP fungal isolates grown on seven different media for antibacterial activity against *E. coli* ATCC 25922 lux. Where possible, we tested each fungus for activity once it had grown to cover 20, 50 and 100% of a 90 mm Petri dish. We measured antibacterial activity as the production of ZOI after incubation with *E. coli* ATCC 25922 lux for 24 h. As the fungal plugs were 6 mm in diameter, a diameter >6 mm indicates the production of a ZOI and, therefore, anti-*E. coli* activity (Fig. 4). We observed strong statistical evidence of the presence of a ZOI differing by phylum (*P*=0.0032) and media (*P*=0.0014) and by the combination of the two (*P*=0.041). There was no evidence of a difference by fungal age.

### Anti-*E. coli* activity varies by fungal phylum

We converted the diameter of the ZOI produced by the fungal isolates into ZOI scores. A diameter of 6 mm, the size of the fungal plug, is given a ZOI score of 0. Zones with a diameter of 7–10 mm are scored as 1, 11–15 mm diameters as 2 and zones with a diameter of >15 mm are scored as 3 (Fig. 4b).

If we define a fungus–medium combination as being active against *E. coli* if the median ZOI score is 1 or higher, then only two fungal isolates, ICMP 17492 (*Umbelopsis ramanniana*) and ICMP 20907 (cf. *Spirosphaera* sp.), were not active (Fig. 4b). The phylum with the most active fungus–medium combinations was the Ascomycota [46/105 (43.8%)], followed by the Basidiomycota [28/84 (33.3%)] and then the Mucoromycota and Mortierellomycota [7/35 (20.0%)] (Fig. 4b).

If we use a more stringent measure of activity, with a fungal isolate required to produce a ZOI with a diameter of >10 mm (a ZOI score≥2), then the number of active ICMP isolates reduces from 30 to 16 (Fig. 4b). The Ascomycota and Mucoromycota have a similar percentage of active fungus–medium combinations, 12.4% (13/105) and 11.4% (4/35), respectively, while the Basidiomycota are the group with the least percentage of active combinations [7.1% (6/84)].

Six ICMP isolates produced ZOI with a diameter of >15 mm (corresponding to a ZOI score of 3): the Ascomycota *Coccomyces radiatus* (ICMP 17340), *Hyaloscypha spinulosa* (ICMP 16865), *L. culmigenum* (ICMP 18328), the Basidiomycota *Agaricales* sp. (ICMP 17554), the Mucoromycota *Cunninghamella echinulata* (ICMP 1083) and the Mortierellomycota *Mortierella* sp. (ICMP 20597) (Fig. 4).

### Differential impact of growth medium on anti*-E. coli *activity

We observed that many of the ICMP fungi displayed differential activity depending on their growth medium (Fig. 4). Using a ZOI score≥1, 24/30 ICMP isolates were active on more than one medium, with seven isolates displaying activity when grown on four or more media. Using a ZOI score≥2, 6/16 ICMP isolates were active on two or more media. The growth medium which resulted in the least anti-*E. coli* activity was CSA. In contrast, growth on OA and PDA resulted in the most activity, with 19/30 and 20/30 ICMP isolates, respectively, exhibiting a ZOI score≥1. This was true when using the more stringent ZOI score of ≥2 (Fig. 4).

### Screening of extracts and fractions from ICMP fungal isolates for anti-*E. coli* activity

Focusing on ICMP isolates which produced activity scores of 2 or above, we prepared extracts from 16 isolates comprising 20 fungus–medium combinations. We further separated these extracts into five fractions, designated F1–F5. As previously described, fraction F1 (100% water) is generally comprised of sugars, while fraction F5 (100% methanol) contains predominantly fatty acids and sterols [19]. Fractions F2, F3 and F4 typically contain the chemical compounds we are most interested in pursuing, with the potential to be bioactive.

**Fig. 5.**
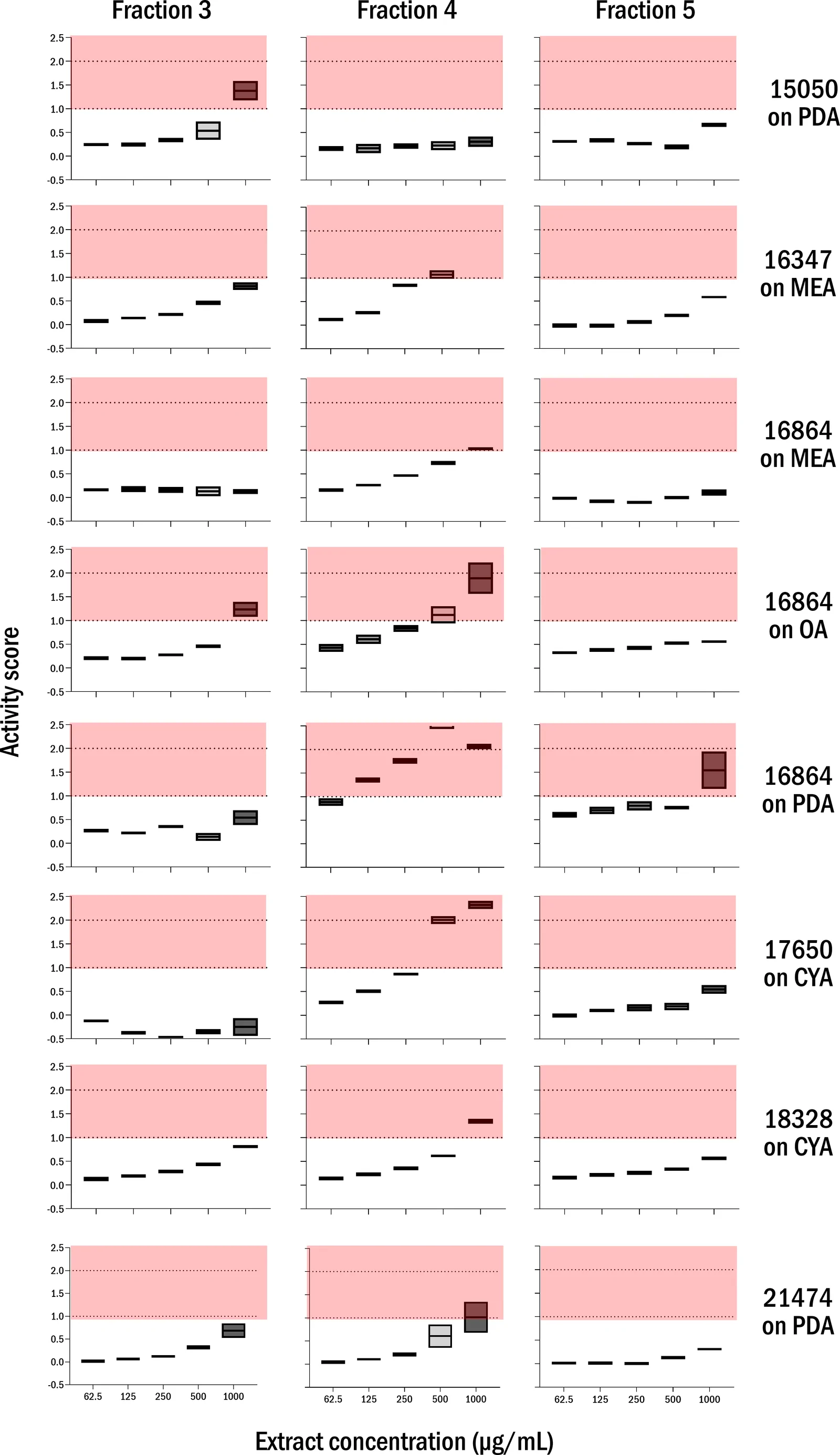
Antibacterial activity of fractions 3–5 from ICMP fungal isolates against *E. coli* 25922 lux. Data are presented as box and whisker plots (with medians) of activity scores. The dotted lines at activity scores of 1 and 2 correspond to a >90% and >99% reduction in bacterial bioluminescence compared to the corresponding no-fraction control, respectively. The red box indicates values above the activity threshold of 1 and so is considered active. Media: Czapek Yeast extract Agar (CYA); Malt Extract Agar (MEA); Oatmeal Agar (OA); Potato Dextrose Agar (PDA). Boxes are upper and lower quartiles with the median shown. The whiskers extend up to 1.5×the inter-quartile range.

We tested the crude extracts and fractions for activity against *E. coli* 25922 lux at concentrations ranging from 0 to 1,000 µg ml^−1^ (Table 3 and Fig. 5). We measured antibacterial activity as reductions in the light output of our bioluminescent *E. coli* strain over 24 h and calculated activity scores as the negative log of the ratio of the Area Under Curve (AUC) values of the fungus-containing measurements and the control measurements. An activity score above 1 corresponds to a >90% reduction in light compared to the control, while an activity score above 2 corresponds to a >99% reduction. We define an extract/fraction as active/antibacterial if the median activity score is above 1.

**Table 3.** MIC as µg ml^−1^ of ICMP fungal crude extracts and fractions (F1–F5) against *E. coli*

Fungus	ICMP	Media*	Fungal age (days)	Coverage (%)	Crude	F1	F2	F3	F4	F5
*Agaricales* sp.	17554	PDA	14	50	>1,000	>1,000	>1,000	>1,000	>1,000	>1,000
*Ce. zonata*	16347	MEA	10	100	>1,000	>1,000	>1,000	>1,000	**500**	>1,000
*Co. radiatus*	17340	OA	32	100	>1,000	>1,000	>1,000	>1,000	>1,000	>1,000
*Cu. echinulata*	1083	CYA	12	100	>1,000	>1,000	>1,000	>1,000	>1,000	>1,000
*Epi. plurivorum*	17650	CYA	10	100	>1,000	>1,000	>1,000	>1,000	**500**	>1,000
*Hy. spinulosa*	16865	PDA	30	20	>1,000	>1,000	>1,000	>1,000	>1,000	>1,000
*Hyp. australianum*	21474	MEA	50	100	>1,000	**1,000**	>1,000	>1,000	**1,000**	>1,000
*La. arvalis*	12896	OA	10	50	>1,000	>1,000	>1,000	>1,000	>1,000	>1,000
*Lau. bellulus*	15050	PDA	9	20	>1,000	>1,000	>1,000	**1,000**	>1,000	**1,000**
*Lopho. culmigenum*	18328	CYA	14	50	>1,000	>1,000	>1,000	>1,000	**1,000**	>1,000
*Mortierella* sp.	20597	MEA	14	50	>1,000	>1,000	>1,000	>1,000	>1,000	>1,000
*Mu. laxorrhixus*	20877	MEA	6	50	>1,000	>1,000	>1,000	>1,000	>1,000	>1,000
		PDA	6	50	>1,000	>1,000	>1,000	>1,000	>1,000	>1,000
*Pe. lycii*	16714	CYA	5	20	>1,000	>1,000	>1,000	>1,000	>1,000	>1,000
		OA	14	100	>1,000	>1,000	>1,000	>1,000	>1,000	>1,000
*Sordariomycetes* sp.	16864	MEA	18	100	>1,000	>1,000	>1,000	>1,000	**1,000**	>1,000
		OA	37	100	>1,000	>1,000	>1,000	**1,000**	**500**	**1,000**
		PDA	18	100	>1,000	>1,000	>1,000	>1,000	**125**	**1,000**
*T. coccinea*	13182	OA	50	10	>1,000	>1,000	>1,000	>1,000	>1,000	>1,000
*Xylariaceae* sp.	16006	CSA	20	100	>1,000	>1,000	>1,000	>1,000	>1,000	>1,000

Key: *Czapek Solution Agar (CSA); Czapek Yeast Extract Agar (CYA); Malt Extract Agar (MEA); Oatmeal Agar (OA); Potato Dextrose Agar (PDA).

### Several ICMP fungal extracts and fractions retain anti-*E. coli* activity

Of the 20 fungus–medium combinations extracted and fractionated, extracts from 6 fungi retained activity with an MIC of at least 1,000 µg ml^−1^ (Table 3). Of these, three had activity below 1,000 µg ml^−1^, all from fraction 4: *Epicoccum plurivorum* ICMP 17650 when grown on CYA (MIC of 500 µg ml^−1^), *Cerrena zonata* ICMP 16347 when grown on MEA (MIC of 500 µg ml^−1^) and *Sordariomycetes* sp. ICMP 16864 when grown on OA (MIC of 125 µg ml^−1^) and PDA (MIC of 500 µg ml^−1^) (Table 3).

### Identification and activity testing of natural products from the active fractions obtained from *E. plurivorum* ICMP 17650 and *Ce. zonata* ICMP 16347

**Fig. 6.**
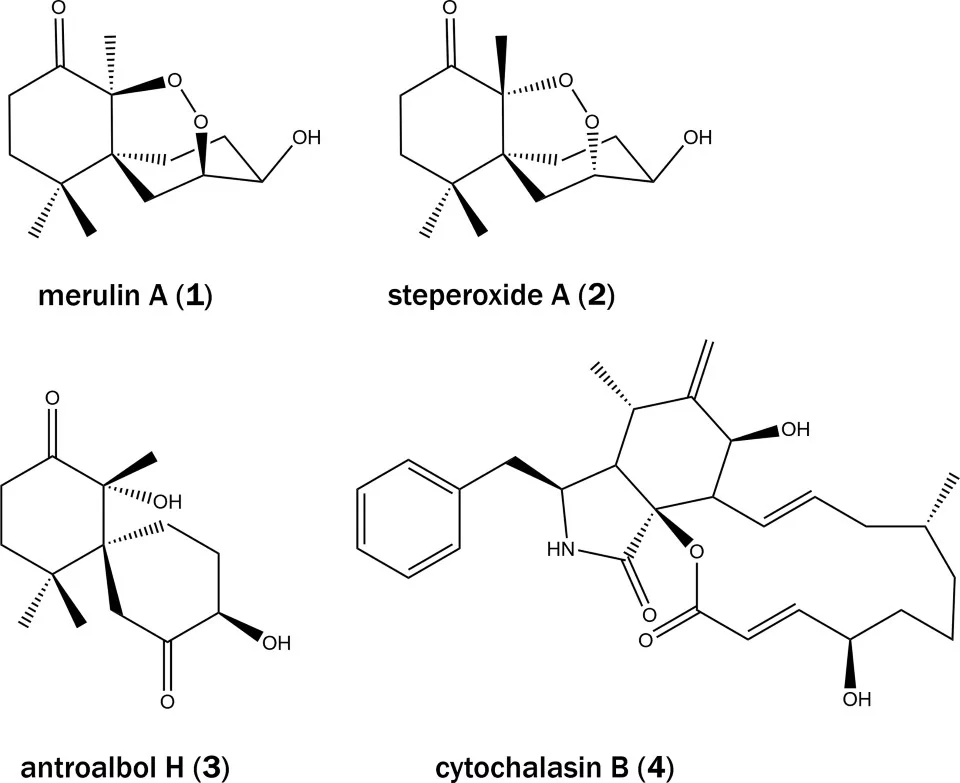
Structures of isolated natural compounds 1–4.

*E. plurivorum* ICMP 17650 and *Ce. zonata* ICMP 16347 were grown on CYA and MEA and extracted with methanol and dichloromethane to afford crude extracts. The crude extracts were subjected to extensive chromatographic methods for purification, including C_8_ reversed-phase column chromatography (H_2_O/MeOH), Sephadex LH-20 (MeOH/5% CH_2_Cl_2_) and silica gel (hexane/EtOAc) chromatography to afford compounds 1–4 (Fig. 6).

A combination of NMR spectroscopy and MS achieved structure elucidation of compounds 1–4. Compounds 1–3, isolated from *Ce. zonata* ICMP 16347, were identified as merulin A [3, 34], steperoxide A [4, 35] and antroalbol H [5, 36]. Compound 4, isolated from *E. plurivorum* ICMP 17650, was identified as cytochalasin B [37].

Before identification, compounds 1–4 were sent to the CO-ADD at The University of Queensland (Australia) to be evaluated for their antimicrobial activity against a panel of bacterial and fungal pathogens: *A. baumannii*, *E. coli*, *K. pneumoniae*, MRSA, *P. aeruginosa*, *C. albicans* and *Cr. neoformans*. None of the compounds exhibited activity when tested at a concentration of 32 µg ml^−1^ (Table 4).

**Table 4.** Antibacterial and antifungal activities of isolated natural compounds as determined by CO-ADD

Natural product	Per cent inhibition at 32 µg ml^−1^*						
	*S. aureus*	*E. coli*	*K. pneumoniae*	*P. aeruginosa*	*A. baumannii*	*C. albicans*	*Cr. neoformans*
Antroalbol H	12.46	4.53	11.23	16.55	10.31	1.76	26.11
Cytochalasin B	−8.45	0.04	1.45	6.71	8.58	3.51	24.68
Merulin A	20.57	9.56	13.69	28.92	13.16	10.13	0.51
Steperoxide A	20.85	23.06	15.99	25.92	20.07	9.77	−19.26

*Data are presented as percentage inhibition (mean of two experiments). *S. aureus* ATCC 43300 (MRSA) with vancomycin (MIC 0.7 µM) as a positive control; *E. coli* ATCC 25922 with colistin (MIC 0.1 µM) as a positive control; *K. pneumoniae* ATCC 700603, *P. aeruginosa* ATCC 27853 and *A. baumannii* ATCC 19606 with colistin (MIC 0.2 µM) as a positive control; *C. albicans* ATCC 90028 with fluconazole (MIC 0.4 µM) as a positive control; *Cr. neoformans* ATCC 208821 with fluconazole (MIC 26 µM) as a positive control.

## Discussion

In this study, we took an OSMAC approach [20] to screen 32 fungi collected from locations across Aotearoa New Zealand, for activity against *E. coli*. Our pipeline involved growing the fungi on seven media and testing for anti-*E. coli* activity. Our rationale for taking this approach is the abundant evidence that nutrient sources influence fungal growth and behaviour, including the production of secondary metabolites [20, 21, 38–40].

For our experiments, we grew fungi on media with different pH and various carbon and nitrogen sources (Table 5). These ranged from the defined (CSA) to the minimal (WA) and included several complex but less chemically defined media made from foods such as oatmeal, potatoes and rice. Our data provide further strong evidence for the impact of media on both fungal growth and bioactivity. One interesting observation from our data is that some fungi grew faster on WA than on CSA and, at times, CYA. We consider WA as a minimal nutrient control, as it is only made up of agarose and agaropectin – though there will also be some trace nutrients carried over from the small PDA plugs used as inocula. We determined that these small plugs did not affect the pH of the media they were placed on, including WA. This suggests that Czapek agar-containing media may suppress the growth of some fungi. These were the only media tested that used sodium nitrate as a nitrogen source, so this may be the reason. We could discern no patterns in the fungi affected, as they belonged to all phyla, with the two most impacted isolates being the saprophytic ascomycete *Lauriomyces bellulus* ICMP 15050 and the polypore basidiomycete *F. maire* ICMP 16416.

**Table 5.** Characteristics of the media used in this study

Media*	Type	Primary carbohydrate source(s)	Primary nitrogen source(s)	Other components	pH
CSA	Defined	Sucrose (glucose and fructose)	Sodium nitrate	Salts, vitamins, trace elements	7.3±0.2
CYA	Complex	Sucrose (glucose and fructose)	Yeast extract, sodium nitrate	Salts, vitamins, trace elements	4.0±0.2
MEA	Complex	Maltose and dextrin	Peptone	Unknown	4.7±0.2
OA	Complex	Starch (amylose and amylopectin)	Amino acids	Unknown	6.0±0.2
PDA	Complex	Glucose	Amino acids	Unknown	5.1±0.2
REA	Complex	Starch (amylose and amylopectin)	Amino acids	Unknown	5.8±0.2
WA	Minimal	Agarose and agaropectin	None	None	7.0±0.2

Key: *Czapek Solution Agar (CSA); Czapek Yeast Extract Agar (CYA); Malt Extract Agar (MEA); Oatmeal Agar (OA); Potato Dextrose Agar (PDA); Rice Extract Agar (REA); Water Agar (WA).

Regarding bioactivity, many fungal isolates were active when grown on more than one medium. So far, we have not yet identified any of the compounds responsible for the anti-*E. coli* activity we observed, so whether these fungi are producing the same bioactive compounds in multiple media is an open question. Fungi possess an array of enzymes that enable them to degrade different organic materials, including cellulose and lignin [41, 42]. This likely provides some redundancy in the biosynthetic pathways required for secondary metabolite synthesis. However, as previously described, abundant evidence shows that fungi can produce different secondary metabolites depending on the available nutrients [20, 21, 38]. For example, we found that fungi grown on OA made more fatty acids, including oleic, palmitic, linoleic and linolenic acids, as quantified by the intensity of NMR signals. In contrast, these same signals were downregulated by growth on MEA (data not shown).

Similar to our results screening ICMP isolates for anti-mycobacterial activity [19], growth on CSA resulted in the least anti-*E. coli* activity, while growth on OA and PDA resulted in the most activity. However, our results caution against restricting future experiments to OA and PDA. If we were to do so, we would likely miss some highly active fungal isolates. For example, *Cu. echinulata* ICMP 1083 produced some of the largest ZOI we observed during this study, but only when grown on CYA.

The ICMP fungi we screened in this project included species of several genera known to produce antimicrobial compounds. For example, Kakoti and colleagues recently isolated cinnabarinic acid from extracts of *Trametes coccinea* mushrooms collected in India [43]. They further showed that cinnabarinic acid could inhibit biofilm formation in *Bacillus subtilis* and *Bacillus cereus*. Chemical extraction and fractionation of a subset of the ICMP isolates, including *T. coccinea*, revealed that much of our observed activity was lost after extraction. This could be because compounds lost activity upon extraction (perhaps due to instability), were produced at too low a concentration to be identified as active in our extract-testing assays or were not produced when fungi were regrown for extraction. Another possibility is that some compounds were acting synergistically *in vivo*, but upon extraction, this synergy was disrupted, perhaps because the compounds were separated into different fractions.

However, we identified extracts from *Ce. zonata*, *E. plurivorum* and an unknown species of *Sordariomycetes* that retained activity. We were able to isolate three known compounds from *Ce. zonata* ICMP 16347 (merulin A/steperoxide B [34, 35], steperoxide A [35] and antroalbol H [36]) and one from *E. plurivorum* ICMP 17650 (cytochalasin B [37]), though none were antibacterial against various human pathogens, including *E. coli*. This suggests that either these compounds were not responsible for the initial activity observed or that they lost activity upon extraction.

Merulin A/steperoxide B was first reported in two papers published in 2010, having been isolated from an unidentified endophytic fungus (XG8D) obtained from the leaves of a species of mangrove (*Xylocarpus granatum*) in Thailand [34] and from the plant pathogenic fungus *Steccherinum ochraceum* from China [35]. Li and colleagues reported that it was unable to inhibit a variety of fungal and bacterial species, including *E. coli*, at a concentration of 50 µg ml^−1^ [44], and our data support this finding. However, Chokpaiboon and colleagues reported that merulin A/steperoxide B was cytotoxic against several human cancer cell lines [34]. More recently, it has been used as a scaffold to develop compounds with activity against the parasite *Trypanosoma brucei*, responsible for human African trypanosomiasis (African sleeping sickness) [45].

Steperoxide A was first reported by Liu and colleagues, having been isolated alongside merulin A/steperoxide B from *St. ochraceum* [35]. From our search of the PubMed, PubChem and SciFinder databases, it does not appear that steperoxide A has previously been tested for antibacterial activity. Similarly, we were unable to find any information for astroalbol H, isolated initially from *Antrodiella albocinnamomea*. It has recently been reported that this compound increases cellular glucose uptake in murine cells, so it may have potential for managing hyperglycaemia [46]. Taken together, our data suggest that none of these compounds were responsible for the activity initially observed for *Ce. zonata* ICMP 16347.

Our data also suggest that the compound isolated from *E. plurivorum* ICMP 17650 – cytochalasin B – was not responsible for the activity initially observed for this fungus. Cytochalasin B is a well-known fungal toxin, widely used in molecular and cell biology, as it inhibits multiple cellular processes [47, 48]. It has previously been shown to be inactive against a variety of bacteria, including *E. coli* [49]; however, a number of other cytochalasins and cytochalasin derivatives do exhibit antibacterial activity [49, 50]. Perhaps in this case, the anti-*E. coli* activity was due to a related cytochalasin compound, which degraded upon or after extraction.

Investigations are ongoing to identify the sources of the anti-*E. coli* activity of *Ce. zonata* ICMP 16347, *E. plurivorum* ICMP 17650 and *Sordariomycetes* sp. ICMP 16864 and to determine whether this may be due to the production of novel bioactive compounds.

## Supplementary material

10.1099/mic.0.001641 Uncited Supplementary Material 1.

## Abbreviations

AUC: area under curve
CO-ADD: Community for Open Antimicrobial Drug Discovery
CSA: Czapek solution agar
CYA: Czapek yeast extract agar
ICMP: International Collection of Microorganisms from Plants
MEA: malt extract agar
MHB: Mueller–Hinton broth II
MRSA: methicillin-resistant *Staphylococcus aureus*
OA: oatmeal agar
OSMAC: one strain-many compounds
PDA: potato dextrose agar
REA: rice extract agar
WA: water agar
ZOI: zone of inhibition
