# Supplementary material for: Using a ‘one strain-many compounds’ approach to screen a collection of diverse fungi from Aotearoa New Zealand for antibacterial activity against Escherichia coli
Source: Microbiology (Reading). 2026 Jan 12;172(1):001641. doi: 10.1099/mic.0.001641 (PMC12825035; doi:10.1099/mic.0.001641)
Supplement: Uncited Supplementary Material 1. [file mic-172-01641-s001.pdf]

Using a 'one strain-many compounds' (OSMAC) approach to screen a collection of diverse fungi from Aotearoa New Zealand for antibacterial activity against *Escherichia coli*

Shara van de Pas[1], Melissa M. Cadelis[1,2], Alexander B.J. Grey[1], Jessica M. Flemming[2], Duckchul Park[3], Thomas Lumley[4], Bevan S. Weir[3], Brent R. Copp[2] and Siouxsie Wiles[1,5*]

[1]Bioluminescent Superbugs Lab, Department of Molecular Medicine and Pathology, Waipapa Taumata Rau - University of Auckland, Auckland, Aotearoa New Zealand

[2]School of Chemical Sciences, Waipapa Taumata Rau - University of Auckland, Auckland, Aotearoa New Zealand

[3]Manaaki Whenua/Landcare Research Ltd., Auckland, Aotearoa New Zealand

[4]Department of Statistics, Waipapa Taumata Rau - University of Auckland, Auckland, Aotearoa New Zealand

[5]Te Pūnaha Matatini: Centre of Research Excellence for Complex Systems and Networks, Aotearoa New Zealand

[*]Correspondence: Dr Siouxsie Wiles (s.wiles@auckland.ac.nz)

Supplementary Methods

Fungal extraction and purification

We grew fungal cultures on solid media at room temperature and then freeze-dried them. We extracted the dry cultures with MeOH (Sigma-Aldrich, New Zealand) for four hours, followed by CH2Cl2 (Sigma-Aldrich, New Zealand) overnight. We concentrated the combined organic extracts under reduced pressure. We subjected the crude extracts to C_8 reversed-phase column chromatography eluting with a gradient of H_2O/MeOH (Sigma-Aldrich, New Zealand) to afford five fractions (F1–F5). Details for each specific isolate are provided in Table S1.

Table S1. Fungal extraction condition details

Isolate	Media	No. plates	Dry weight (g)	MeOH (L)	Ch_2Cl_2 (L)	Oil produced (g)
1083	CYA	25	163.98	1.0	1.0	6.81
12896	OA	23	23.12	0.5	0.5	6.43
13182	OA	20	29.88	0.45	0.45	2.70
15050	PDA	45	39.47	0.35	0.35	10.17
16006	CSA	43	35.54	0.75	0.75	13.98
16347	CYA	19	15.88	0.4	0.4	2.70
	MEA	53	67.33	1.0	1.0	4.76
16714	CYA	40	29.02	0.8	0.8	7.70
	OA	37	36.12	0.7	0.7	3.88
16864	OA	40	41.57	0.75	0.75	6.03
	PDA	32	16.12	1.0	1.0	2.00
	MEA	18	7.99	0.5	0.5	0.82
16865	PDA	59	70.67	1.0	1.0	2.38
17340	OA	44	58.22	0.75	0.75	6.81
17554	PDA	39	21.17	1.0	1.0	6.51
17650	CYA	37	28.68	0.75	0.75	4.00
18328	CYA	28	25.28	0.5	0.5	5.05
20597	MEA	22	11.76	0.5	0.5	4.29
20877	MEA	27	21.50	0.5	0.5	1.69
	PDA	22	11.21	0.5	0.5	0.81
21474	MEA	38	22.18	1.0	1.0	8.26

Key: CYA, Czapek Yeast Agar; OA, Oatmeal Agar; PDA, Potato Dextrose Agar; CSA, Czapek Solution Agar; MEA, Malt Extract Agar.

Fraction F3 obtained from ICMP 16347 grown on CYA was subjected to purification by Sephadex LH-20, eluting with MeOH, to afford seven fractions (A1–A7). Further purification of fraction A3 by silica gel column chromatography, eluting with gradient n-hexane/EtOAc, afforded antroalbol H (4.62 mg). Purification of A2 by silica gel column chromatography, eluting with gradient n-

hexane/EtOAc, followed by trituration with methanol afforded merulin A (9.90 mg) and steperoxide A (3.54 mg).

Fraction F4 obtained from ICMP 16347 grown on MEA was purified by diol-bonded silica gel column chromatography, eluting with gradient n-hexane/EtOAc to afford four fractions (A1–A4). Further purification of fraction A1 by silica gel column chromatography, eluting with gradient n-hexane/EtOAc, afforded steperoxide A (30.13 mg). Purification of A2 by silica gel column chromatography, eluting with gradient n-hexane/EtOAc, afforded merulin A (7.16 mg).

Fraction F4 obtained from ICMP 17650 grown on CYA was triturated with dichloromethane, and the precipitate was purified by silica gel column chromatography, eluting with gradient n-hexane/EtOAc, to afford cytochalasin B (4) (23.19 mg).

Supplementary Data

Table S2. Physical characteristics of the fungal isolates used in this study.

Fungus	ICMP number	Media	Form	Elevation	Margin	Colour
Agaricales sp.	17554	CSA	Circular	Flat	Filiform	Transparent
		CYA	Circular	Raised	Filiform	White
		MEA	Circular	Raised	Filiform	Cream
		OA	Circular	Raised	Filiform	White
		PDA	Circular	Raised	Filiform	Cream
		REA	Circular	Raised	Filiform	Transparent
		WA	Circular	Flat	Filiform	Transparent
Annulohypoxylon sp.	18216	CSA	Circular	Raised	Filiform	White
		CYA	Circular	Raised	Filiform	White
		MEA	Circular	Raised	Filiform	White
		OA	Circular	Raised	Filiform	White
		PDA	Circular	Raised	Filiform	White
		REA	Circular	Raised	Filiform	White
		WA	Circular	Raised	Filiform	White
Cerrena zonata	16347	CSA	Circular	Raised	Filiform	White
		CYA	Circular	Raised	Filiform	White
		MEA	Circular	Raised	Filiform	White
		OA	Circular	Raised	Filiform	White
		PDA	Circular	Raised	Filiform	White

		REA	Circular	Raised	Filiform	Transparent
		WA	Circular	Raised	Filiform	Transparent
Coccomyces radiatus	17340	CSA	Circular	Flat	Filiform	Transparent
		CYA	Irregular	Raised	Undulate	Yellow
		MEA	Irregular	Raised	Undulate	Yellow
		OA	Circular	Raised	Entire	Yellow
		PDA	Irregular	Raised	Entire	Yellow
		REA	Circular	Flat	Filiform	Transparent
		WA	Circular	Flat	Filiform	Transparent
Conchomyces bursiformis	16580	CSA	Filamentous	Flat	Filiform	Transparent
		CYA	Filamentous	Flat	Filiform	White
		MEA	Irregular	Flat	Filiform	Peach
		OA	Filamentous	Flat	Filiform	White
		PDA	Irregular	Flat	Filiform	Peach
		REA	Filamentous	Flat	Filiform	Opaque
		WA	Filamentous	Flat	Filiform	Transparent
Cunninghamella echinulate	1083	CSA	Circular	Flat	Filiform	Transparent
		CYA	Circular	Raised	Filiform	White
		MEA	Circular	Raised	Filiform	White
		OA	Circular	Raised	Filiform	White
		PDA	Circular	Raised	Filiform	White
		REA	Circular	Flat	Undulate	Transparent
		WA	Circular	Flat	Filiform	Transparent
Epicoccum plurivorum	17650	CSA	Rhizoid	Flat	Filiform	Light brown
		CYA	Circular	Raised	Filiform	Brown
		MEA	Irregular	Raised	Filiform	Brown
		OA	Circular	Raised	Filiform	Moggy
		PDA	Irregular	Raised	Filiform	Brown
		REA	Rhizoid	Flat	Filiform	Transparent
		WA	Rhizoid	Flat	Filiform	Transparent
Fomitopsis maire	16416	CSA	Circular	Flat	Filiform	White
		CYA	Circular	Raised	Entire	White
		MEA	Circular	Raised	Entire	White
		OA	Circular	Raised	Entire	White
		PDA	Circular	Raised	Entire	White
		REA	Circular	Flat	Entire	Opaque
		WA	Circular	Flat	Filiform	Transparent
Helicoon pluriseptatum	16276	CSA	Filamentous	Flat	Filiform	Light brown
		CYA	Circular	Raised	Undulate	Dark brown
		MEA	Circular	Raised	Entire	Black
		OA	Circular	Raised	Undulate	Dark brown

		PDA	Circular	Raised	Undulate	Black
		REA	Filamentous	Flat	Filiform	Light brown
		WA	Filamentous	Flat	Filiform	Light brown
Hohenbuehelia nothofaginea	16703	CSA	Filamentous	Flat	Filiform	Transparent
		CYA	Circular	Flat	Filiform	Cream
		MEA	Filamentous	Flat	Filiform	White
		OA	Circular	Flat	Entire	White
		PDA	Filamentous	Flat	Filiform	White
		REA	Filamentous	Flat	Filiform	Transparent
		WA	Irregular	Flat	Filiform	Transparent
Hyaloscypha spinulosa	16865	CSA	Rhizoid	Flat	Filiform	Cream
		CYA	Circular	Flat	Entire	Cream
		MEA	Circular	Flat	Entire	Cream
		OA	Circular	Flat	Entire	Cream
		PDA	Circular	Raised	Entire	Cream
		REA	Circular	Flat	Entire	Opaque
		WA	Circular	Flat	Entire	Transparent
Hymenotorrendiella brevisetosa	18823	CSA	Circular	Flat	Lobate	Purple
		CYA	Circular	Flat	Lobate	Purple
		MEA	Circular	Flat	Entire	Brown
		OA	Circular	Flat	Entire	Opaque
		PDA	Circular	Flat	Entire	Brown
		REA	Circular	Flat	Entire	Opaque
		WA	Circular	Flat	Entire	Opaque
Hypholoma australianum	21474	CSA	Circular	Flat	Filiform	Opaque
		CYA	Circular	Flat	Entire	Cream
		MEA	Irregular	Flat	Entire	White
		OA	Circular	Flat	Entire	White
		PDA	Irregular	Flat	Entire	White
		REA	Rhizoid	Flat	Filiform	Opaque
		WA	Rhizoid	Flat	Entire	Opaque
Hypoderma cordylines	16705	CSA	Circular	Flat	Filiform	Transparent
		CYA	Circular	Flat	Filiform	Dark brown
		MEA	Circular	Raised	Filiform	Light brown
		OA	Circular	Flat	Filiform	Brown
		PDA	Circular	Raised	Filiform	Light brown
		REA	Circular	Flat	Filiform	Transparent
		WA	Circular	Flat	Filiform	Transparent
Laetisaria arvalis	12896	CSA	Rhizoid	Flat	Filiform	Cream
		CYA	Filamentous	Raised	Filiform	Peach
		MEA	Filamentous	Raised	Filiform	Cream

		OA	Filamentous	Raised	Filiform	Cream
		PDA	Filamentous	Raised	Filiform	Cream
		REA	Filamentous	Flat	Filiform	Cream
		WA	Rhizoid	Flat	Filiform	Transparent
Lanzia allantospora	15649	CSA	Filamentous	Raised	Filiform	Transparent
		CYA	Circular	Raised	Entire	Cream
		MEA	Filamentous	Raised	Undulate	Brown
		OA	Filamentous	Raised	Filiform	Brownish-grey
		PDA	Filamentous	Raised	Entire	Brown
		REA	Filamentous	Raised	Filiform	Transparent
		WA	Filamentous	Raised	Filiform	Transparent
Lauriomyces bellulus	15050	CSA	Circular	Flat	Entire	Opaque
		CYA	Circular	Flat	Entire	Cream
		MEA	Circular	Flat	Entire	Cream
		OA	Circular	Flat	Entire	Light brown
		PDA	Circular	Flat	Entire	Cream
		REA	Circular	Flat	Filiform	Opaque
		WA	Circular	Flat	Entire	Transparent
Lentinellus pulvinulus	16586	CSA	Transparent	Filamentous	Flat	Filiform
		CYA	Cream	Circular	Flat	Entire
		MEA	Cream	Filamentous	Flat	Filiform
		OA	Transparent	Filamentous	Flat	Filiform
		PDA	Cream	Filamentous	Flat	Filiform
		REA	Transparent	Filamentous	Flat	Filiform
		WA	Transparent	Rhizoid	Flat	Filiform
Linnemannia elongata	17447	CSA	Rhizoid	Flat	Filiform	Transparent
		CYA	Circular	Raised	Filiform	Transparent
		MEA	Circular	Raised	Filiform	White
		OA	Circular	Raised	Filiform	Transparent
		PDA	Circular	Raised	Filiform	White
		REA	Circular	Flat	Filiform	Transparent
		WA	Circular	Flat	Filiform	Transparent
Lophiotrema sp.	20449	CSA	Circular	Flat	Filiform	Opaque
		CYA	Circular	Flat	Filiform	Brown
		MEA	Irregular	Raised	Entire	Black
		OA	Circular	Flat	Filiform	Brown
		PDA	Circular	Raised	Entire	Black
		REA	Circular	Flat	Filiform	Opaque
		WA	Circular	Flat	Filiform	Transparent
	18328	CSA	Circular	Flat	Filiform	Brown

Lophodermium culmigenum		CYA	Circular	Flat	Filiform	Brown
		MEA	Circular	Flat	Filiform	Brown
		OA	Circular	Flat	Filiform	Brown
		PDA	Circular	Flat	Filiform	Brown
		REA	Circular	Flat	Filiform	Brown
		WA	Circular	Flat	Filiform	Brown
Mortierella sp.	20597	CSA	Filamentous	Raised	Filiform	Transparent
		CYA	Filamentous	Raised	Filiform	Transparent
		MEA	Filamentous	Raised	Filiform	White
		OA	Filamentous	Raised	Filiform	White
		PDA	Filamentous	Raised	Filiform	White
		REA	Filamentous	Raised	Filiform	Transparent
		WA	Filamentous	Raised	Filiform	Transparent
Mucor laxorrhixus	20877	CSA	Rhizoid	Flat	Filiform	Grey
		CYA	Filamentous	Flat	Filiform	Grey
		MEA	Filamentous	Flat	Filiform	Grey
		OA	Filamentous	Flat	Filiform	Grey
		PDA	Filamentous	Flat	Filiform	Grey
		REA	Filamentous	Flat	Filiform	Grey
		WA	Rhizoid	Flat	Filiform	Grey
Peniophora lycii	16714	CSA	Filamentous	Raised	Filiform	Transparent
		CYA	Filamentous	Raised	Filiform	Cream
		MEA	Filamentous	Raised	Filiform	Cream
		OA	Filamentous	Raised	Filiform	Cream
		PDA	Filamentous	Raised	Filiform	Cream
		REA	Filamentous	Raised	Filiform	Transparent
		WA	Filamentous	Raised	Filiform	Transparent
Pleohelicoon richonis	16226	CSA	Circular	Umbonate	Filiform	Brown
		CYA	Circular	Flat	Filiform	Brown
		MEA	Circular	Umbonate	Filiform	Brown
		OA	Circular	Raised	Entire	Brown
		PDA	Circular	Umbonate	Undulate	Brown
		REA	Circular	Flat	Filiform	Transparent
		WA	Circular	Flat	Filiform	Transparent
Rigidoporus concrescens	18193	CSA	Filamentous	Raised	Filiform	White
		CYA	Filamentous	Raised	Filiform	White
		MEA	Filamentous	Raised	Filiform	White
		OA	Filamentous	Raised	Filiform	White
		PDA	Filamentous	Raised	Filiform	White
		REA	Filamentous	Raised	Filiform	White
		WA	Filamentous	Raised	Filiform	White

Sordariomycetes sp.	16864	CSA	Rhizoid	Flat	Filiform	Green
		CYA	Circular	Flat	Entire	Dark green
		MEA	Circular	Flat	Filiform	Green
		OA	Circular	Flat	Filiform	Dark green
		PDA	Circular	Flat	Filiform	Green
		REA	Circular	Flat	Filiform	Green
		WA	Rhizoid	Flat	Filiform	Transparent
cf. *Spirosphaera sp.*	20907	CSA	Circular	Flat	Filiform	Transparent
		CYA	Circular	Flat	Entire	Brown
		MEA	Circular	Raised	Undulate	Brownish grey
		OA	Circular	Flat	Entire	Dark brown
		PDA	Circular	Raised	Undulate	Dark brown
		REA	Circular	Flat	Filiform	Transparent
		WA	Circular	Flat	Entire	Transparent
Trametes coccinea	13182	CSA	Circular	Flat	Filiform	Transparent
		CYA	Circular	Flat	Filiform	Opaque
		MEA	Circular	Flat	Filiform	White
		OA	Circular	Flat	Filiform	Peach
		PDA	Circular	Flat	Filiform	Peach
		REA	Circular	Flat	Filiform	Transparent
		WA	Rhizoid	Flat	Filiform	Transparent
Trechispora stevensonii	17555	CSA	Circular	Flat	Filiform	Opaque
		CYA	Circular	Flat	Entire	Opaque
		MEA	Circular	Flat	Filiform	Cream
		OA	Circular	Flat	Entire	White
		PDA	Circular	Flat	Filiform	Cream
		REA	Circular	Flat	Filiform	Opaque
		WA	Circular	Flat	Filiform	Transparent
Umbelopsis ramanniana	17492	CSA	Rhizoid	Flat	Filiform	Transparent
		CYA	Circular	Flat	Entire	Pink
		MEA	Circular	Flat	Filiform	Pink
		OA	Circular	Flat	Filiform	Dark pink
		PDA	Circular	Flat	Filiform	Dark pink
		REA	Circular	Flat	Filiform	Light pink
		WA	Rhizoid	Flat	Filiform	Transparent
Xylariaceae sp.	16006	CSA	Irregular	Raised	Filiform	Opaque
		CYA	Circular	Raised	Filiform	White
		MEA	Circular	Umbonate	Lobate	White
		OA	Circular	Raised	Filiform	White
		PDA	Circular	Umbonate	Entire	White

	REA	Circular	Raised	Filiform	Opaque
	WA	Irregular	Flat	Filiform	Transparent

Key: CSA, Czapek Solution Agar; CYA, Czapek Yeast Extract Agar; MEA, Malt Extract Agar; OA, Oatmeal Agar; PDA, Potato Dextrose Agar; REA, Rice Extract Agar; WA, Water Agar.
